# Ziyuglycoside I promotes wound healing via GPR34-mediated activation of group 3 innate lymphoid cells

**DOI:** 10.1186/s13020-026-01483-0

**Published:** 2026-08-24

**Authors:** Kewei Wang, Qingya Sun, Gang Wang, Jiayan Zhang, Yehuang Wang, Bin Jiang, Yang Zhang

**Affiliations:** 1 Colorectal Surgery Center, Nanjing Hospital of Chinese Medicine Affiliated to Nanjing University of Chinese Medicine, Nanjing City, Jiangsu Province China; 2 School of Integrated Medicine, Nanjing University of Chinese Medicine, Nanjing City, Jiangsu Province China; 3 General Surgery Department, Nanjing Hospital of Chinese Medicine Affiliated to Nanjing University of Chinese Medicine, Nanjing City, Jiangsu Province China

**Keywords:** Ziyuglycoside I, Wound healing, GPR34, ILC3s

## Abstract

**Background:**

*Sanguisorba officinalis* L., a traditional Chinese medicine, has been frequently utilized to promote the healing of burns and scalds. Ziyuglycoside I (ZG-I), a bioactive compound isolated from the roots of S. officinalis L., exhibits significant properties for wrinkle care, antioxidant activity, and antiviral effects. This study aims to explore the underlying mechanisms by which ZG-I promotes wound healing.

**Methods:**

The efficacy of the treatment was evaluated by monitoring wound closure rates and conducting histological analyses to assess inflammation and re-epithelialization. Immunohistochemistry, utilizing Ki67, α-SMA, and Vimentin antibodies, further characterized the epidermal and dermal repair processes. In vitro, Group 3 innate lymphoid cells (ILC3s) were co-cultured with primary mouse skin fibroblasts, and their proliferation and migration were assessed. The expression of GPR34, secretion of IL-22, and downstream pathways were evaluated. The mechanism was confirmed by knocking down GPR34 in ILC3s. Finally, molecular docking studies, CETSA, and MST experiments validated the interaction between ZG-I and GPR34.

**Results:**

ZG-I significantly accelerated wound closure in vivo, accompanied by reduced inflammation and immune cell infiltration, enhanced re-epithelialization, and improved collagen fiber organization. Mechanistically, ZG-I upregulated the expression of GPR34 in ILC3s within the wound tissues and elevated the secretion of IL-22. Furthermore, ZG-I dose-dependently enhanced fibroblast viability, proliferation, and migration, while promoting GPR34 and IL-22 expression in ILC3s in vitro. The knockdown of GPR34 in ILC3s reversed these effects and inhibited downstream signaling pathways, including STAT3, AKT, and MAPK. These results establish that ZG-I promotes wound healing by activating GPR34 in ILC3s, which triggers the AKT and MAPK pathways to enhance STAT3 phosphorylation and IL-22 production.

**Conclusion:**

Our results indicate that ZG-I has the activity of promoting wound healing, and GPR34 can serve as a therapeutic target for ZG-I in the treatment of skin injury-related diseases.

**Graphical Abstract:**

**Figure:**
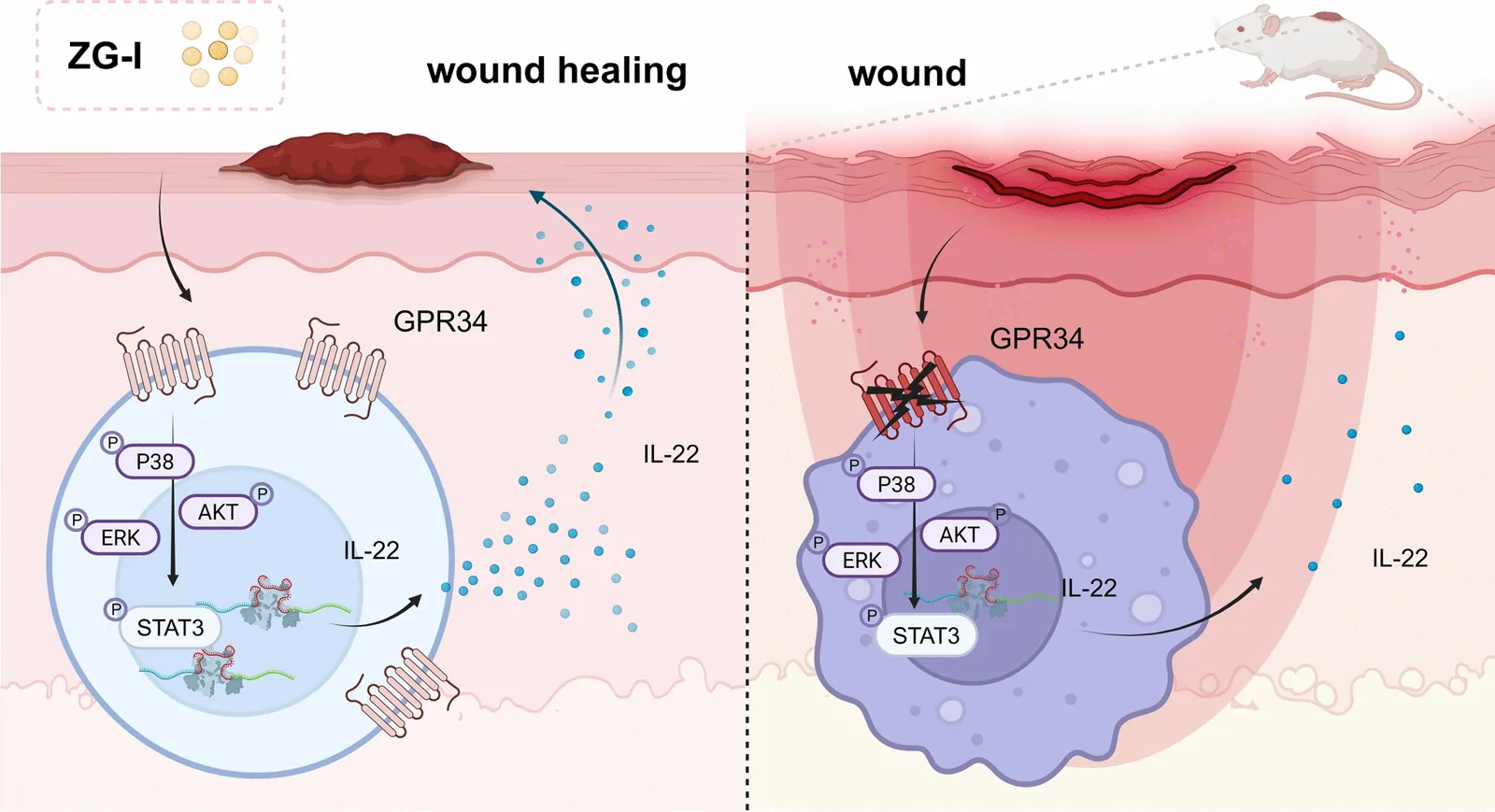

## Introduction

Wounds represent a prevalent clinical concern due to exposure to environmental stressors. As the primary defense mechanism against external insults, the epidermis frequently experiences disruptions in its continuity. Injuries necessitate swift repair to preserve barrier integrity, sustain protective functions, and mitigate infection risks [1, 3]. When damage extends beyond the epidermal layers, affecting basal and differentiated cells as well as deeper structures such as the dermis and extracellular matrix (ECM), it triggers hemorrhage and initiates coagulation through vasoconstriction and platelet aggregation. Concurrently, inflammatory cells, including neutrophils and macrophages, along with fibroblasts, are recruited to the site, where proteolytic enzymes break down necrotic debris and initiate tissue remodeling [2–5].

Innate lymphoid cells (ILCs) are derived from lymphoid progenitors and have been shown to perform diverse functions in immunity and tissue homeostasis [6]. ILCs are classified into three distinct subsets based on their cytokine profiles and transcriptional regulators, with a predominant presence in the stroma of mucosal tissues [7, 8]. Group 3 innate lymphoid cells (ILC3s), characterized by the expression of retinoic acid receptor-related orphan receptor γ (RORγ), play critical roles in maintaining epithelial tissue homeostasis and regeneration [9]. However, their physiological contribution to normal skin tissue repair remains largely unexplored. G Protein-Coupled Receptor 34 (GPR34) serves as a damage-detecting receptor that is specifically expressed by ILC3s, initiating tissue repair mechanisms upon detecting of apoptotic neutrophils. Research indicates that the absence of Gpr34 or its downstream signaling pathways, particularly the AKT and ERK pathways, in ILC3s restricts the production of IL-22 and impairs wound healing during colon and skin injuries [10]. Tissue injury typically initiates a complex cytokine network that orchestrates post-traumatic repair processes. Among these mediators, IL-22, a member of the type II cytokine family, is rapidly secreted to modulate immune responses against various epithelial insults. Growing experimental evidence underscores its dual functional capacity: while it contributes to inflammatory cascades, IL-22 also plays critical roles in epithelial tissue regeneration and barrier restoration. The dynamic involvement of this cytokine in both pro-inflammatory signaling and tissue repair mechanisms underscores its significance in the physiology of wound healing [11–13].

*Sanguisorba officinalis* L., a traditional Chinese medicine, has been utilized for over 2000 years in China to promote hemostasis and treat burns and scalds [14, 15]. The herb's roots contain a broad spectrum of phenolic terpenoid compounds that primarily exhibit anti-inflammatory and anti-infective effects [16]. Ziyuglycoside I (ZG-I), one of the bioactive compounds isolated from the roots of *S. officinalis* L., demonstrates significant efficacy in wrinkle care, as well as antioxidant and antiviral activities [17, 18]. Understanding the pathogenesis and pharmacological treatment of wound healing is essential for developing effective treatment strategies. However, the mechanisms by which ZG-I influences wound healing remain unclear and warrant further investigation both in vitro and in vivo. In the present study, we demonstrated that ZG-I accelerates wound healing in mice, a finding that was further validated in vitro. Mechanistically, ZG-I enhances the expression of GPR34, thereby improving the sensing of LysoPS released during tissue injury. This activation triggers downstream signaling cascades, including AKT and MAPK pathways, ultimately leading to the activation of ILC3s and subsequent production of IL-22. Collectively, these processes promote wound repair, highlighting the critical role of ILC3 in tissue repair.

## Materials and methods

### Materials and reagents

The Ziyuglycoside I (B20294, HPLC ≥ 98%, CAS: 35286-58-9) was acquired from Shanghai yuanye Bio-Technology Co., Ltd. (Shanghai, China). Recombinant Human Epidermal Growth Factor Gel was purchased from Guilin Pavay Gene Pharmaceutical Co. Ltd. (Guilin, China). The specific formula is shown in Table 1.

**Table 1:** Formulation of ZG-I containing cream

Phase	Component	Mass fraction (%)	
		Base emulsion	Ziyuglycoside I (0. 1%)
A	Isopropyl myristate	3.5	3.5
	Polydimethylsiloxane	3.5	3.5
	Liquid paraffin	1.2	1.2
	Glyceryl stearatese	1.2	1.2
	Cetearyl alcohol	2	2
	Sucrose stearate ester	1.5	1.5
B	Glycerin	3.5	3.5
	Butanediol	3.5	3.5
	Xanthan gum	0.15	0.15
	EDTA-2Na	0.03	0.03
	Pure water	To 100	To 100
C	Methyl isothiazolinone	0.01	0.01
D	Active ingredient	0	0.1

Mix the components of phase A and phase B uniformly. After heating to 80 ℃, slowly pour phase A into phase B. After homogenizing (5000 rpm/min, 5 min), stir and cool down to 40 ℃, then add phases C and D in sequence, stir evenly

This formulation follows standard dermatological cream preparation protocols, ensuring homogeneity, stability, and bioavailability of the active ingredient (ZG-I). All components comply with pharmacopeial standards for topical dosage forms

### Animals

BALB/c male mice (weight: 18–25 g) were bought from Beijing Vital River Laboratory Animal Technology Co., Ltd. (Beijing, China). All mice were raised under specific pathogen-free conditions at 24 ± 1 °C with a 12 h light: dark cycle for 1 week to facilitate adaptation. Food and water were available without restriction. All procedures were conducted according to Regulations on Experimental Animal Management. Additionally, the welfare and testing protocols were rigorously aligned with the Laboratory Animal Care and Ethics Guidelines set forth by Nanjing University of Chinese Medicine (Approval No. 202504A095). The 3R principles in laboratory animals were strictly followed in this study: reduction, replacement, and refinement.

### Animal models of wound healing

Wound healing was evaluated in vivo using wound models. After intraperitoneal injection of pentobarbital sodium for anesthesia, the fur on the backs of mice was removed with depilatory cream. A circle (diameter of 10 mm) was marked along the midline of the spinal column on the mouse’s back, followed by creating a full-thickness skin wound using a biopsy punch. After hemostasis with cotton swabs, mice were randomly assigned to 6 groups (n = 12): Model, Base (base cream), Low (0.1 mg/kg), Medium (0.2 mg/kg), High (0.4 mg/kg), and Positive. From the second day post-operation, mice were administered medication twice daily. After 14 days of treatment, the mice were euthanized, and relevant tissue samples were collected for subsequent experiments.

### Isolation and culture of murine group 3 innate lymphoid cells

The colon was excised and placed in sterile petri dish containing ice-cold PBS (Solarbio, P1020). Mucosal layer was promptly isolated using fine forceps under a stereomicroscope. Mucosal tissues were minced into approximately 1 mm fragments and transferred to HBSS (Solarbio, H1045) supplemented with 5% FBS (Gibco, A5256701) and 5 mM EDTA (Solarbio, E8030). The suspension was vortexed at 250 rpm for 20 min, followed by discarding the supernatant. Post the second incubation, samples were filtered and transferred to EP tubes for further mincing. Tissue fragments were then digested in HBSS containing collagenase D (Merck, 11088858001; 1.5 g/L), DNase I (Merck, 10104159001; 40 mg/L), and dispase II (Gibco, 17105041; 3 g/L) at 37 °C with constant rotation (250 rpm) for 15 min. Then, cells were collected into 5 mL conical tubes, centrifuged at 4 °C (1500 rpm, 10 min), resuspended in precooled HBSS/FBS buffer, and kept on ice until use. ILCs were stimulated with IL-2 (10 ng/mL; R and D Systems), IL-7 (10 ng/mL; R and D Systems), IL-1β (20 ng/mL; BioLgend), and IL-23 (20 ng/mL; Thermo Fisher Scientific) for 10 days to induce ILC3 differentiation.

### Isolation and culture of murine primary dermal fibroblasts

Mice were euthanized by cervical dislocation, followed by immersion in 75% ethanol for 5 min. Skin was collected into precooled PBS buffer containing double antibiotics (Biosharp, BL505A). Adipose and vascular tissues were removed and washed with PBS. Trypsin digestion (Biosharp, BL501A) was performed for 1 h to separate epidermis from dermis. The dermal fraction was minced in a petri dish, resuspended in trypsin solution, and transferred to a centrifuge tube for further incubation at 37 °C in water for 1 h. Digestion was terminated by adding DMEM complete medium (Biosharp, BL304A). Supernatant was discarded. Washed tissues were placed in a 6 cm culture dish with 2 mL medium overnight. The next day, medium volume was increased to 5 mL for continuous cultivation. When substantial cell migration occurred, tissue fragments were removed, and fresh medium was replaced for subsequent expansion.

### GPR34 inhibition in vitro

ILC3 cells were seeded 24 h before transfection at a confluency of 70–80%. The cells were then treated with either GRP34 siRNA (100 nM) (si-GPR34 sequence: CCUACUGAUAAUCCUCUCAUA) or control siRNA, mixed with EZ Trans transfection reagent (high efficiency) (AC04L092, Life-ilab). After 5 h of incubation, the cells were washed and cultured overnight. Subsequently, cells were harvested over 48 h post-transfection for expression level validation [19].

### ELISA analysis

The tissues were homogenized in PBS. The supernatant obtained was utilized as the substrate, following the guidelines supplied by the kit manufacturer. Serial dilutions of the standard were prepared at concentrations of 500 pg/ml, 250 pg/ml, 125 pg/ml, 62.5 pg/ml, 31.25 pg/ml, 15.63 pg/ml, and 7.81 pg/ml. Aliquots of these diluted standards were added to designated wells on the microplate, while blank wells received only the diluent buffer. Test samples were dispensed into corresponding sample wells. Subsequently, detection antibodies, streptavidin conjugate solution, signal enhancer reagent, and chromogenic substrate were sequentially introduced according to the protocol. Absorbance values were measured at 450 nm wavelength using a SpectraMax microplate reader (Molecular Devices, San Jose, CA). The resulting optical density data were used for quantitative analysis based on the established standard curve. MULTISCIENCES (Hangzhou, China) provided the Mouse IL-22 High Sensitivity ELISA Kit (EK222HS).

### Western blot analysis

After homogenizing the tissue, the tissue lysate was prepared with the buffer containing protease and phosphatase inhibitors, plus PMSF, and incubated at 4 °C. The Bio-Rad protein assay was employed to measure the concentration in the supernatant. Samples were boiled with loading buffer for 5 min. Proteins were separated by 15% SDS-PAGE, then transferred to PVDF membranes. Membranes were incubated with primary antibodies at 4 °C overnight. After washed with TBST, membranes were incubated with HRP-conjugated antibodies for 60 min. Reactive bands were detected through the ECL reagent and quantified with ImageJ software.

Cells were lysed using RIPA buffer (Beyotime, P0013B) supplemented with a protease inhibitor cocktail (Beyotime, P1045). Total proteins were extracted, separated by SDS-PAGE, and transferred onto a PVDF membrane. The membrane was blocked with 5% non-fat dry milk over 2 h, followed by incubation at 4 °C with primary antibodies. HRP-conjugated secondary antibodies and ECL substrate (Biosharp, BL520A) were used for protein detection.

Tubulin (AF7011, dilution 1:5000), GPR34 (DF4972 dilution 1:1000), P-P38 (AF4001 dilution 1:1000), STAT3 (AF6294 dilution 1:1000), and P-STAT3 (AF3293 dilution 1:1000) was purchased from Affinity Biosciences. Proteintech Group, Inc. provided the Anti-rabbit IgG (SA00001-2, dilution 1:5000), GAPDH (10494-1-AP dilution 1:5000), and Anti-rabbit IgG (H + L) (SA00001-2 dilution 1:5000). Cell Signaling Technology (CST) provided the AKT (#9272 dilution 1:1000), P-AKT (#4060 dilution 1:1000), ERK (#4695 dilution 1:1000), P-ERK (#4370 dilution 1:1000), P38 (#9212 dilution 1:1000), p-JNK (#4668 dilution 1:1000), and JNK (#67096 dilution 1:1000).

### Flow cytometry protocol for immune cell isolation and staining

Following humane euthanasia, full-thickness back skin containing wounds and adjacent tissue (≈8 mm × 8 mm) was excised, placed on a nitrocellulose membrane, and trimmed to remove subcutaneous fat and muscle. The tissue was rinsed with PBS, minced into 4–5 small pieces, and digested in 700 µL of enzyme cocktail (Liberase TL [0.35 mg/mL], Collagenase D [3 mg/mL], and DNase I [0.1 mg/mL] in RPMI-1640 medium) at 37 °C with shaking (1,400 rpm) for 40 min. The digestate was filtered through a 70 µm cell strainer, and red blood cells were lysed using Red Blood Cell Lysis Buffer (Beyotime, C3702) for 1–2 min at room temperature. Cells were washed with ice-cold flow cytometry loading buffer (PBS containing 1% BSA and 2 mM EDTA) and centrifuged at 400 × g for 5 min at 4 °C. The cell pellet was resuspended in 200 µL of ice-cold flow cytometry loading buffer, and after counting, 0.5 × 10^6^ to 1 × 10^6^ cells were transferred to a 1.5 mL EP tube for staining (all steps performed protected from light). For intracellular cytokine detection, cells were pre-stimulated with PMA (50 ng/mL) and Ionomycin (500 ng/mL) for 4 h, with secretion blocked by Brefeldin A (1 μg/mL).

For extracellular staining, cells were centrifuged at 400 × g for 5 min at 4 °C, and the supernatant was discarded. The pellet was resuspended in 50 µL of blocking buffer (flow cytometry buffer containing Anti-mouse CD16/CD32 [Beyotime, C1755] and 1% BSA) and incubated at 4 °C for 20 min. After centrifugation, cells were stained with 50 µL of extracellular antibody cocktail (APC Anti-Mouse CD45 Antibody [Elabscience, E-AB-F1136E], FITC anti-mouse Lineage Cocktail [Biolegend, 133302], PE/Cyanine5 Anti-Mouse CD90.2 Antibody [Elabscience, E-AB-F1094G]) at 4 °C for 30 min protected from light, followed by three washes with ice-cold flow cytometry loading buffer. For intracellular staining, the Transcription Factor Staining Buffer Set (Elabscience, E-CK-A108) was used. Cells were fixed with 100 µL of fixation buffer at room temperature for 30 min, washed, and then incubated with 50 µL of intracellular antibody cocktail (PE-Cyanine7 RORγt Antibody [eBioscience, 25-6981-82], PE anti-mouse IL-22 Antibody [Biolegend, 516404], PE anti-STAT3 Phospho (Tyr705) Antibody [Biolegend, 651004]) diluted in the kit's nuclear permeabilization buffer at 4 °C for 1 h protected from light. After washing, cells were resuspended in 400 µL of ice-cold flow cytometry loading buffer, filtered through a 35 µm cell strainer, and transferred to flow cytometry tubes for acquisition. Gating strategy excluded cell debris, doublets, and dead cells sequentially, followed by identification of target cells as CD45⁺Lin⁻CD90.2⁺RORγt⁺IL-22⁺.

### Histology and immunohistochemistry analysis

The skin was fixed in 4% paraformaldehyde overnight, followed by dehydration via a graded series of ethanol. Subsequently, samples were embedded in paraffin, sectioned, and stained with hematoxylin and eosin. For immunohistochemical analysis, paraffin sections were deparaffinized with xylene and ethanol, after which endogenous peroxidase activity was blocked by incubating the sections in a 3% H_2_O_2_ solution. Following this, the slides were blocked with a 10% goat serum mixture and incubated with antibodies. Finally, the slides were treated with a DAB substrate solution to visualize the antibody staining, followed by counterstaining with hematoxylin for enhanced visualization and quantification. Vimentin (#5741, dilution 1:200), α-Smooth Muscle Actin (D4K9N) XP® Rabbit mAb (#19245, dilution 1:400) were acquired from Cell Signaling Technology (CST); KI67 (ab15580, dilution 1:1000) was acquired from Abcam plc (NASDAQ: ABCM).

### Immunofluorescence assay

The skin tissues were fixed in 4% paraformaldehyde for 24 h. Target areas were sectioned into 30 μm slices and washed 3 times in 1xPBS. The slices were incubated in Citrate Antigen Retrieval Solution using a prosthetic device. After incubating with the primary antibody and the secondary antibody, the slices were mounted on slides. Nuclei were stained with DAPI for half hour. Washing 3 times with 1xPBS. The samples were finally blocked with 95% glycerol. Visualization was performed using a confocal scanning microscope (Nikon, Tokyo, Japan).

The fibroblasts were collected, counted, and seeded into well plates. Cells were immobilized with 4% paraformaldehyde, followed by permeabilization using 0.3% Triton X-100 for an additional 15-min incubation. After washing with PBS, cells were blocked with 10% goat serum (Solarbio, SL038). Followed by incubation at 4 °C overnight with primary antibodies. Samples were equilibrated at room temperature for 30 min. Then incubated with secondary antibodies for 1 h. Nuclei were counterstained by incubating with DAPI solution (Solarbio, C0065) at room temperature for 10 min. Finally, samples were mounted using an anti-fluorescence quenching mounting medium (Solarbio, S2100) and observed under a fluorescence microscope for imaging and data recording.

Invitrogen provided the Anti-Hu/Mo ROR gamma (14-6988-82, dilution 1:400) and GPR34 Polyclonal Antibody (PA5-102096, dilution 1:100). Phospho-Stat3 (Tyr705) (D3A7) XP^®^ Rabbit mAb (#9145, dilution 1:100) were acquired from Cell Signaling Technology (CST).

### MTT

Skin fibroblasts were seeded into 96-well plates, incubated for 24 h. After removing the supernatant, 5 mg/mL MTT solution (MCE, HY-15924) was adde, followed by an additional 4 h of culture. Termination of incubation was performed by carefully discarding the medium from all wells. Subsequently, 100 μL of DMSO was introduced into each well, followed by shaking at 100 rpm for 10 min on an orbital shaker. Finally, absorbance values at 490 nm were measured using a microplate reader.

### 5-ethynyl-2’-deoxyuridine (EDU) cell proliferation assay

Skin fibroblasts were seeded into appropriate well plates and incubated overnight. EDU labeling was performed according to the manufacturer’s protocol (UE, Cat# C6043): cells were pulsed with 10 µM EDU for 2 h at 37 °C, followed by fixation with 4% paraformaldehyde (Biosharp, BL539A) for 30 min. Subsequently, permeabilization was conducted using PBS containing 0.5% Triton X-100 (Solarbio, T8202) for 15 min. After adding sufficient staining solution, nuclear counterstaining was achieved by Hoechst 33342 incubation in dark for 10 min. Fluorescence microscopy was employed to visualize and document experimental outcomes. UElandy (Suzhou) Co., Ltd. provided the YF^®^ 488 Click-iT EdU (C6043).

### Transwell cell migration and invasion assay

ILC3 cells pretreated with drugs and primary dermal fibroblasts were collected separately. The ILC3 were seeded into the lower chamber of a Transwell system, while the primary dermal fibroblasts were plated in the upper chamber. After culturing for 24 h, cells were fixed with 4% paraformaldehyde for 15 min, followed by staining with 0.1% crystal violet solution (Solarbio, G1063) for 30 min. Subsequently, samples were observed under a microscope and documented.

### Molecular docking

The 2D structure of Ziyuglycoside I was retrieved in SDF format from the PubChem database. The ligand was imported into Discovery Studio, where hydrogen atoms were added, and energy minimization was performed to optimize the molecular geometry. The 3D structure of mouse GPR34 was obtained from the AlphaFold Database in PDB format. The file was submitted to the POCASA web server (https://g6altair.sci.hokudai.ac.jp/g6/service/pocasa/) with default parameters to predict potential active pockets. The protein structure was then processed in Discovery Studio: hydrogen atoms were added, and non-protein molecules were removed. The predicted active pocket coordinates from POCASA were used to define the binding site in Discovery Studio. The optimized ligand was docked into the pocket using the Dock Ligands (LibDock) protocol. The LibDockScore for each docking pose was recorded to evaluate binding affinity.

### Experimental procedure for cellular thermal shift assay (CETSA)

The CETSA was performed to evaluate the direct binding of ZG-I to isolated ILC3 cells. ILC3 cells were cultured to 70–80% confluence and divided into two groups. Control group treated with DMSO, and experimental group treated with ZG-I. After incubation, cells were washed twice with pre-cooled PBS (containing protease inhibitors), detached using trypsin, and collected by centrifugation. The cell pellets were resuspended in PBS (without protease inhibitors), counted, and aliquoted into PCR tubes. A temperature gradient (55, 60, 65, 70, 75, 80, and 85 °C) was applied using a PCR instrument: samples were heated to each target temperature for 3 min, cooled at room temperature for 3 min, and then placed on ice to prevent protein refolding. After thermal challenge, cells were lysed with 50 μL of pre-cooled lysis buffer, and soluble proteins were extracted by centrifugation at 20,000 × g for 20 min at 4 °C. The supernatants (containing soluble non-denatured proteins) were subjected to Western blot analysis using standard protocols to detect target protein stability.

### Microscale Thermophoresis (MST) binding assay

The binding affinity between Ziyuglycoside I (CAS: 35286-58-9) and GPR34 protein was determined using Microscale Thermophoresis (MST). Based on the molecular docking results that predicted Ziyuglycoside I binding to the active pocket of GPR34, both the wild-type (WT-GPR34) and a mutant protein with key interacting amino acids substituted (MUT-GPR34) were utilized to validate the binding specificity.

The target proteins (WT- or MUT-GPR34) were labeled with Pico-RED fluorescent dye. Ziyuglycoside I was serially diluted in a concentration gradient ranging from 1000 to 0.001 μM. A constant concentration of 50 nM labeled protein was incubated with each concentration of the ligand for 10 min at room temperature. The mixtures were then loaded into standard glass capillaries. MST measurements were performed on a NanoTemper Monolith NT.115 instrument. The dissociation constant (Kd) values were determined and analyzed using the MO.affinity Analysis software (v2.3). A lower Kd value for WT-GPR34 indicates more stable binding between Ziyuglycoside I and the protein, and a significant decrease in affinity for MUT-GPR34 would confirm the critical role of the predicted amino acid residues in the active pocket for the interaction.

### Statistical analysis

All statistical data were expressed as mean ± standard deviation (SD). One-way analysis of variance (ANOVA) was carried out using GraphPad Prism version 9.5 software (GraphPad Software, CA, USA), followed by Tukey’s post hoc test. Each experiment was repeated three times. Values of *P* < 0.05 were considered significant, and values of *P* < 0.01 were considered markedly significant.

## Results

### Ziyuglycoside I exhibit wound-healing promoting activity

**Fig. 1:**
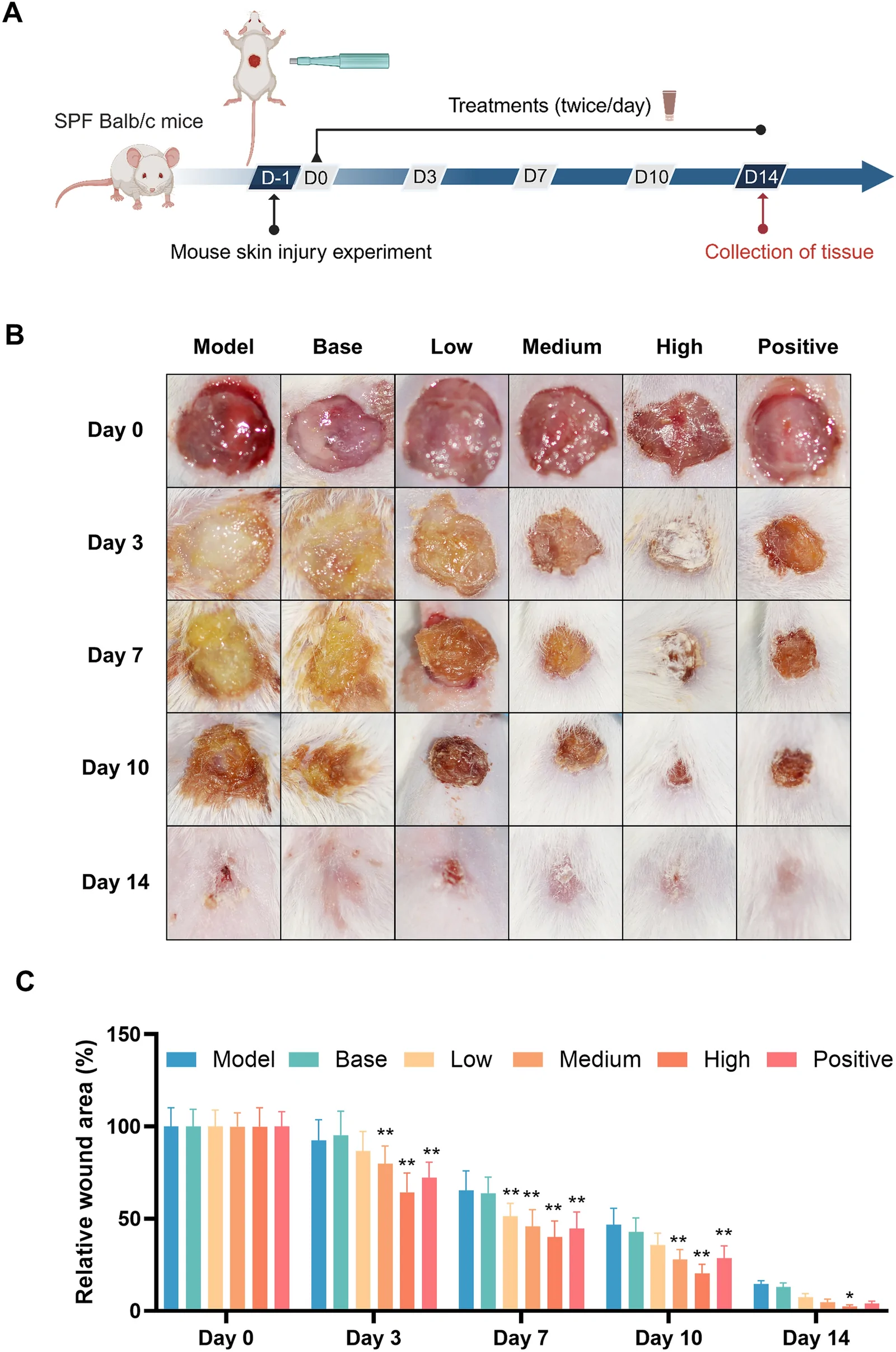
ZG-I accelerated mouse skin wound closure. **A** Schedule of experimental procedures. **B** Representative wound images from mice were taken at specified intervals. **C** The percentage of wound area (relative to the original wound area on day 0). n = 12, ^*^P < 0.05, ^**^P < 0.01, vs Base group

**Fig. 2:**
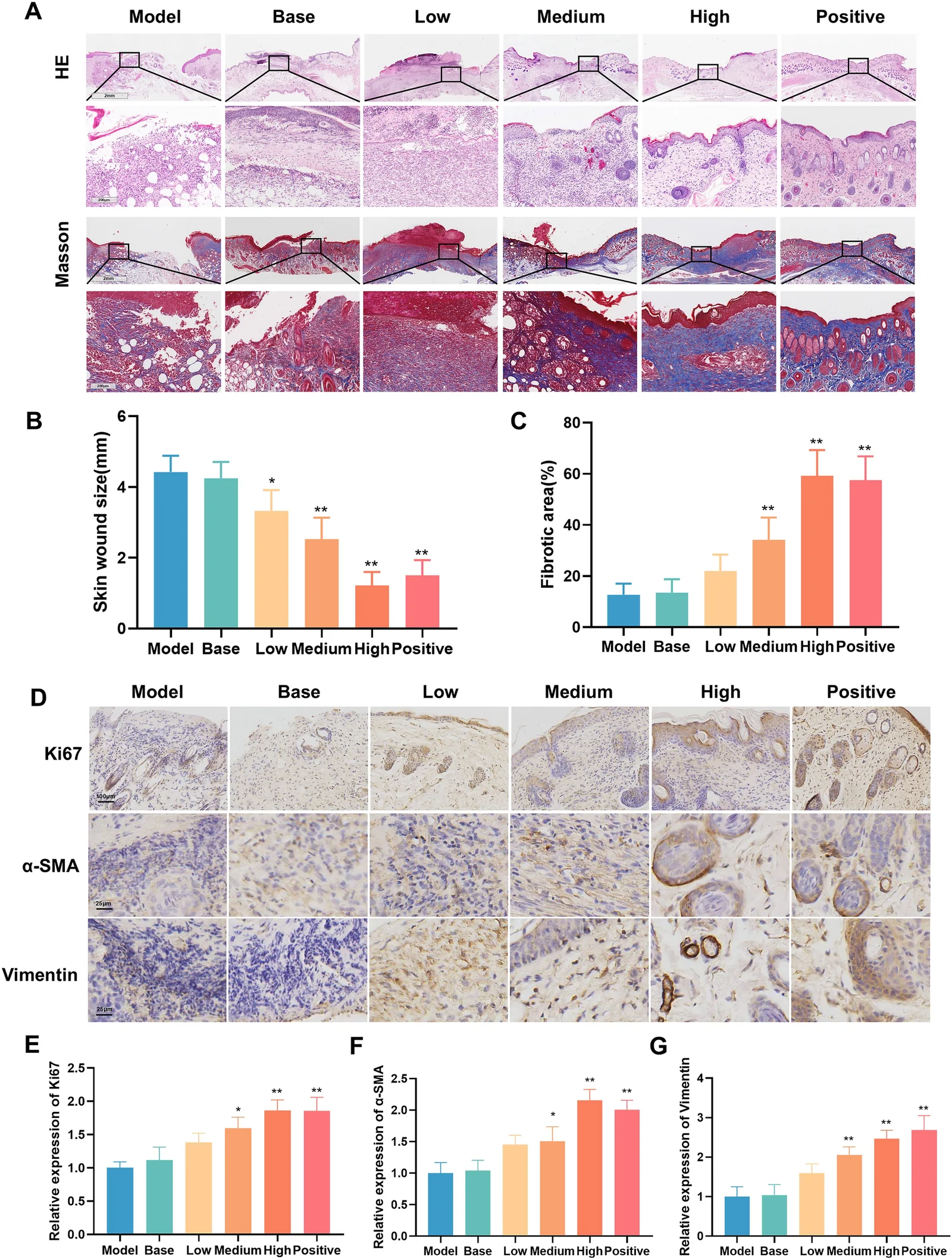
Effects of ZG-I on wound healing. **A** Representative histological images of HE and Masson staining. n = 6. **B**, **C** Effect of ZG-I on skin wound size and fibrotic area. n = 6. **D** Immunohistochemical images for vimentin (Scale bar: 25 μm), α-SMA (Scale bar: 25 μm), and Ki67 (Scale bar: 100 μm) antibodies. n = 3. **E**–**G** Relative expression of Ki67, α-SMA, and Vimentin. n = 3. ^*^P < 0.05, ^**^P < 0.01, vs Base group

To confirm the wound healing activities of ZG-I, we made full-thickness excisional injury in mice dorsal skin (10 mm in diameter). The rate of wound closure was used to assess the efficacy of potential wound healing agent. The procedure schedule was illustrated in Fig. 1A–C shows that topical application of ZG-I enables faster tissue recovery, compared to the Base group. Histopathological examination of wound morphology demonstrated markedly decreased inflammatory responses and immune cell infiltration, alongside accelerated re-epithelialization in the High and Positive groups compared to the Base group. Masson staining also revealed dense and well-organized collagen fiber deposition around the incision site treated with ZG-I (Fig. 2A–C). Immunohistochemical analysis of Ki67, α-SMA, and Vimentin further demonstrated accelerated migration, maturation, and proliferation of epidermal and dermal cells in the ZG-I and Positive groups relative to the Base group, which are processes critical for effective wound healing (Fig. 2D–G).

### Ziyuglycoside I promote the expression of GPR34 and IL-22

**Fig. 3:**
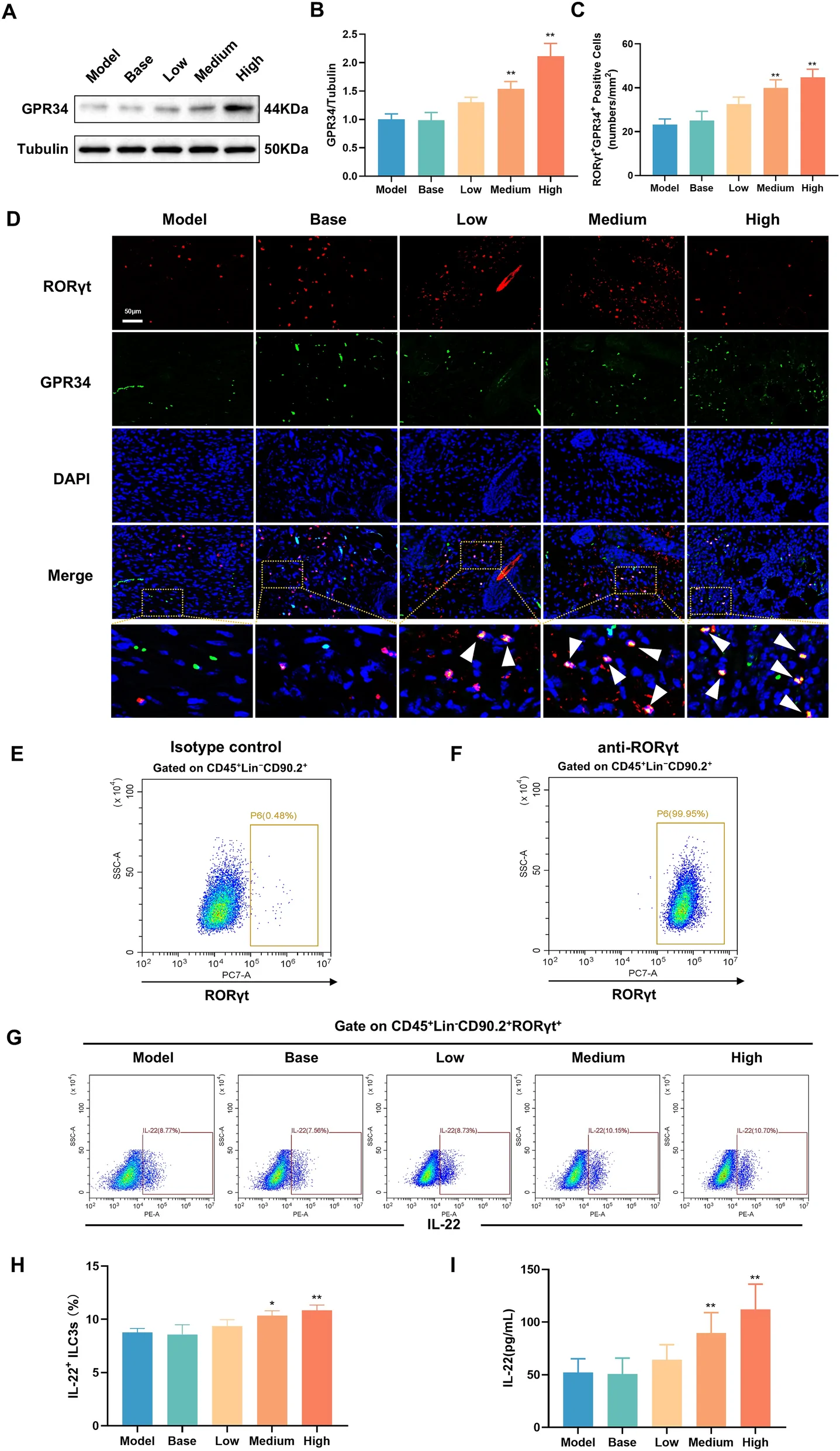
Effect of ZG-I on the expression of GPR34 and IL-22. **A**, **B** The effects of increasing concentrations of ZG-I on GPR34 levels were investigated by western blot analysis and quantified by densitometry. n = 3. **C**, **D** Immunofluorescence images for GPR34 (green), RORγt (red), and DAPI (blue). Representative images are shown (Scale bar: 50 μm). n = 3. **E**, **F** The purity identification results of isolated ILC3s. **G**, **H** Representative flow cytometry plots demonstrated a change of IL-22 (gated on CD45^+^Lin^−^CD90.2^+^RORγt^+^) in mice skin. n = 3. **I** Detection of IL-22 expression by ELISA. n = 6. ^*^P < 0.05, ^**^P < 0.01, vs Base group

GPR34 is a damage-sensing receptor expressed by ILC3 [10]. ILC3 can orchestrate wound healing and tissue repair through the secretion of IL-22 [6]. To investigate how ZG-I promotes wound healing, we conducted the following experiments. WB analysis reveals a significant elevation in GPR34 levels within the skin of wounds from the High group (Fig. 3A, B). Furthermore Fig. 3C, D, show that the quantity of RORyt^+^GPR34^+^ cells is markedly greater than that observed in the Base group, suggesting that ZG-I promotes the activation of ILC3. Subsequently, we examined the immune signaling in wounded regions. Following the purity identification of ILC3s (Fig. 3E, F), flow cytometry analysis revealed that the proportion of IL-22^+^ ILC3s exhibited a clear dose-dependent upward trend with increasing treatment doses, compared to the Base group (Fig. 3G, H). Consistently, ELISA results demonstrated an elevated secretion of IL-22 in skin tissues from the High group (Fig. 3I). Therefore, ZG-I may promote the activation of ILC3 by enhancing GPR34 expression in ILC3s, subsequently driving IL-22 secretion to facilitate epithelial cell proliferation and repair.

### Ziyuglycoside I activate the downstream pathway of GPR34 to regulate the activation of ILC3

**Fig. 4:**
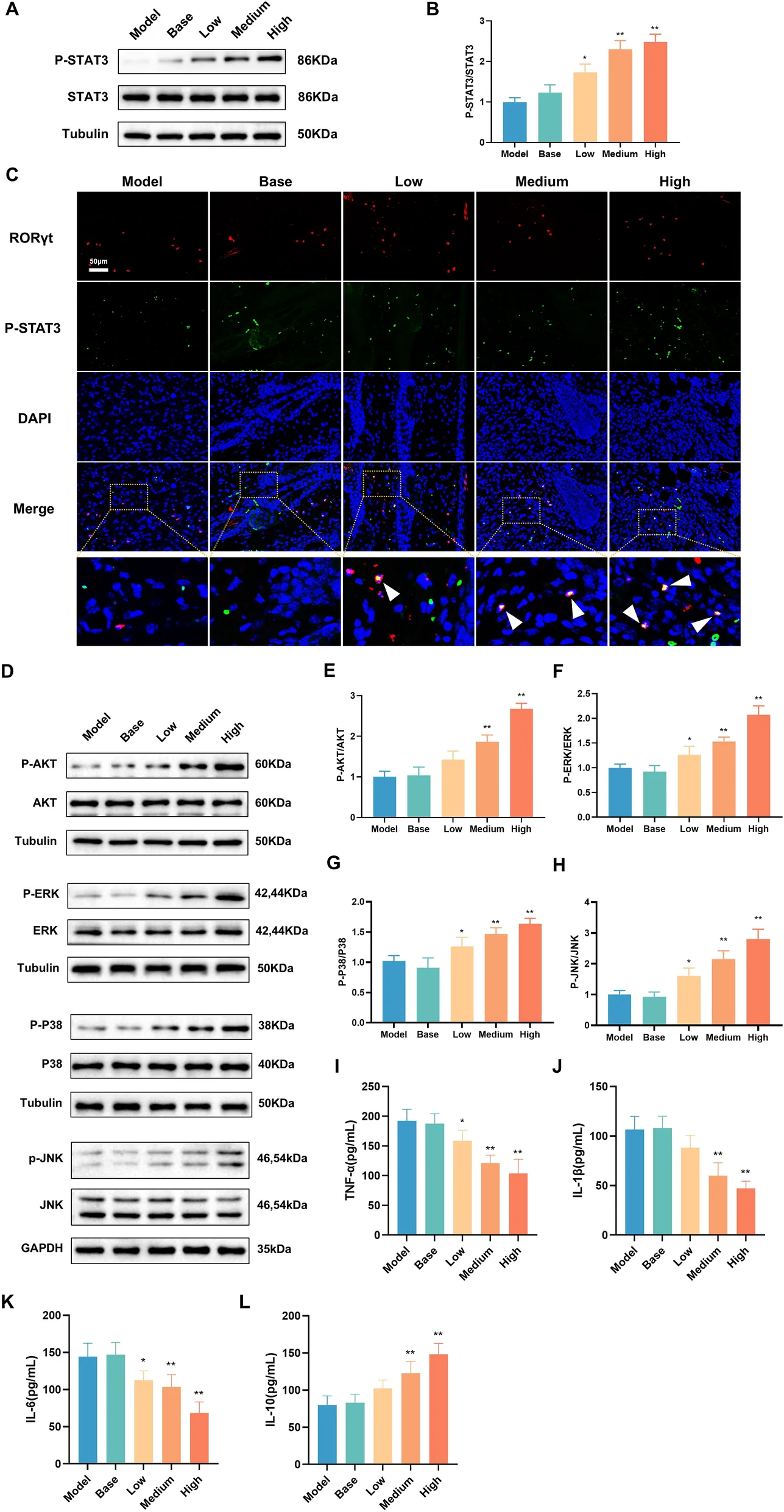
Ziyuglycoside I activate the downstream pathway of GPR34. **A**, **B** The effects of increasing concentrations of ZG-I on P-STAT3 and STAT3 levels were investigated by western blot analysis and quantified by densitometry. n = 3. **C** Immunofluorescence images for P-STAT3 (green), RORγt (red), and DAPI (blue). Representative images are shown (Scale bar: 50 μm). n = 3. **D**–**H** The effects of increasing concentrations of ZG-I on AKT, P-AKT, ERK, P-ERK, P38, P-P38, P-JNK, and JNK levels were investigated by western blot analysis and quantified by densitometry. n = 3. **I**–**L** The expression of TNF-α, IL-1β, IL-6, and IL-10 was detected by ELISA. n = 6. ^*^P < 0.05, ^**^P < 0.01, vs Base group

Subsequently, we investigated the downstream signaling mechanisms of GPR34 associated with ILC3 activation, where the transcription factor STAT3 modulates IL22 production within these cells [20]. In Fig. 4A, B, we examined the expression of STAT3. Compared with the Base group, the expression of P-STAT3/STAT3 was notably increased in High group. Furthermore, the immunofluorescence image in Fig. 4C shows co-localization of RORγt with P-STAT3, indicating that STAT3 is significantly activated in ILC3s. GPR34 is a Gi/o protein-coupled receptor that activates classic signaling cascades of the associated G-protein family. Thus, we measured the expression levels and phosphorylation status of AKT, ERK, P38, and JNK in the mice skin, and obtained noteworthy results (Fig. 4D–H). The findings indicate that P38, AKT, and ERK signaling pathways function downstream of GPR34 to regulate STAT3 activation and IL-22 expression in ILC3s. We also detected the levels of pro-inflammatory and anti-inflammatory factors in wound skin. The results demonstrated that increasing doses of ZG-I led to a significant decrease in pro-inflammatory factors (IL-1β, IL-6, TNF-α) and a marked increase in the anti-inflammatory factor IL-10, suggesting its potential role in modulating the inflammatory response during wound healing (Fig. 4I–L).

### Ziyuglycoside I promote the expression of GPR34 and IL-22 in vitro

**Fig. 5:**
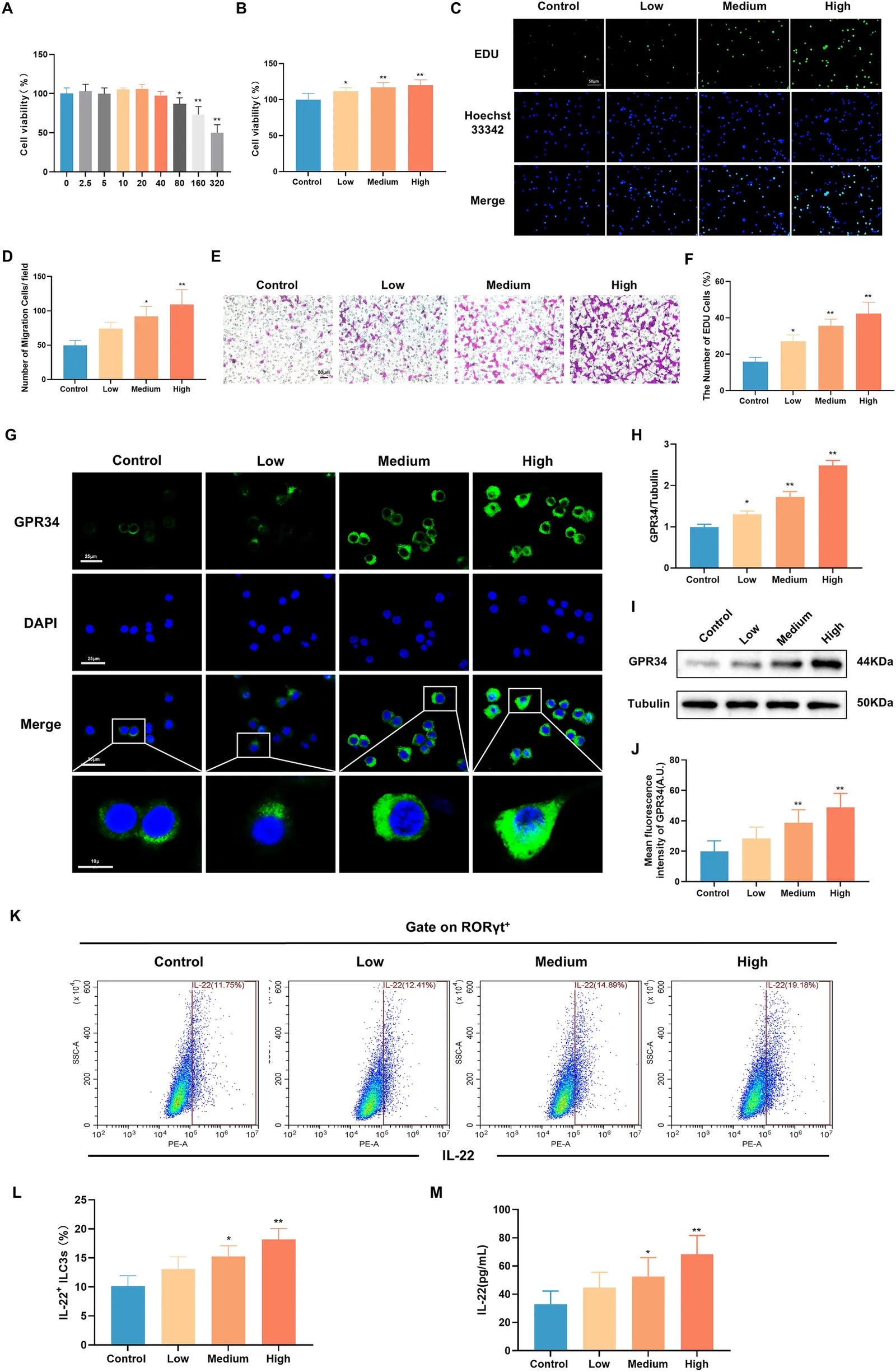
Ziyuglycoside I promote the expression of GPR34 and IL-22 in vitro. **A**, **B** MTT assay for cell viability (%). n = 6. **C**, **D** Representative immunofluorescence images and quantitative analysis for EDU (green), and Hoechst 33342 (blue). n = 3. **E**, **F** Representative images and quantitative analysis of Transwell assay for mouse primary dermal fibroblasts. n = 3. **G**, **H** Immunofluorescence images and quantitative analysis for GPR34 (green) and DAPI (blue). Representative images are shown (Scale bar: 25 μm). n = 3. **I**, **J** The effects of increasing concentrations of ZG-I on GPR34 levels were investigated by western blot analysis and quantified by densitometry. n = 3. **K**, **L** Representative flow cytometry plots demonstrated change of IL-22 (gated on RORγt^+^). n = 3. **M** Detection of IL-22 expression by ELISA. n = 6. ^*^P < 0.05, ^**^P < 0.01, vs Control group

**Fig. 6:**
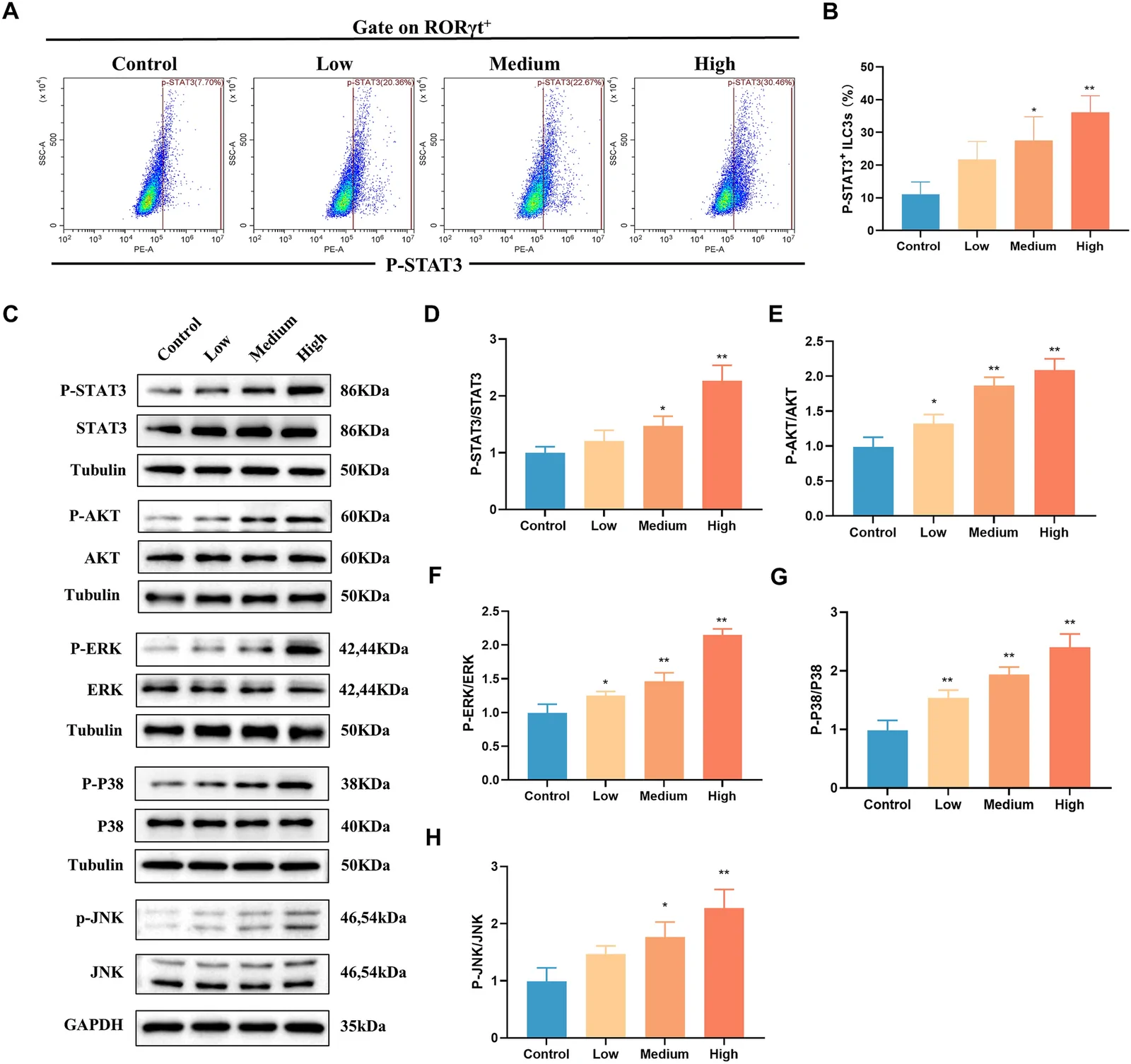
Ziyuglycoside I activate the downstream pathway of GPR34 in vitro. **A**, **B** Representative flow cytometry plots demonstrated change of P-STAT3 (gated on RORγt^+^). n = 3. **C**–**H** The effects of increasing concentrations of ZG-I on STAT3, P-STAT3, AKT, P-AKT, ERK, P-ERK, P38, P-P38, P-JNK, and JNK levels were investigated by western blot analysis and quantified by densitometry. n = 3. ^*^P < 0.05, ^**^P < 0.01, vs Control group

Following the demonstration that ZG-I effectively facilitates epithelial cell proliferation and repair in vivo, we investigated whether ZG-I could also promote cell proliferation in vitro. After determining the maximum non-toxic concentration of ZG-I on ILC3 cells, three concentrations were selected for subsequent experiments (Fig. 5A). The gradient concentration of ZG-I was screened and used to treat ILC3s. Subsequently, these cells were co-cultured with primary mouse skin fibroblasts. Upon detection of the viability of the primary mouse skin fibroblasts, it was observed that ZG-I significantly enhanced their cellular viability (Fig. 5B). Similarly, EDU⁺ cells counts and the migration cells/field showed a significant increase after drug administration (Fig. 5C–F). Subsequently, we detected the expression of GPR34 in ILC3s treated with different concentrations of drugs using WB and immunofluorescence assays. It showed that the expression of GPR34 increased with the rise of ZG-I concentration (Fig. 5G–J). Consistent with the in vivo results, flow cytometry analysis showed that treatment of ILC3 cells with ZG-1 significantly increased the intracellular levels of IL-22 (Fig. 5K, L) and P-STAT3 (Fig. 6A, B). Enzyme-linked immunosorbent assay further demonstrated that the drug treatment significantly elevated the secretion of IL-22 (Fig. 5M). In ILC3s, the expression levels and phosphorylation status of STAT3, AKT, ERK, P38, and JNK were also upregulated as expected following drug administration (Fig. 6C–H).

### Ziyuglycoside I promote cell proliferation and wound healing in vitro by regulating the downstream pathway of GPR34

**Fig. 7:**
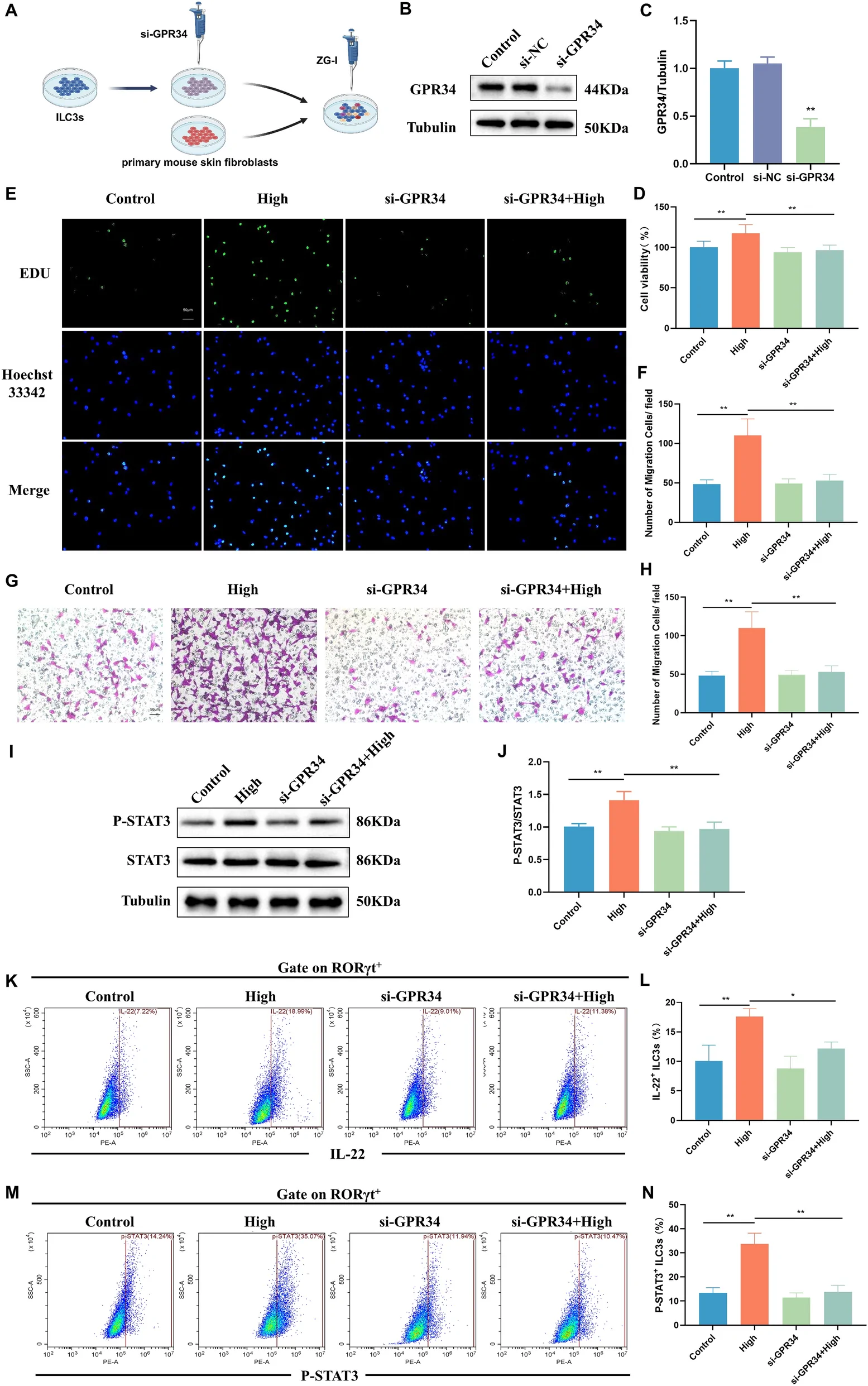
Knockdown of GPR34 reverse the effect of Ziyuglycoside I. **A** Schedule of experimental procedures. **B**, **C** Western blot analysis for GPR34 in ILC3s. n = 3. **D** MTT assay for cell viability (%). n = 6. **E**, **F** Representative immunofluorescence images and quantitative analysis for EDU (green) and Hoechst 33342 (blue). n = 3. **G**, **H** Representative images and quantitative analysis of Transwell assay. n = 3. **I**, **J** Western blot analysis for P-STAT3 and STAT3. **K**–**N** Representative flow cytometry plots demonstrated change of IL-22 (gated on RORγt^+^) and P-STAT3 (gated on RORγt^+^). n = 3. ^*^P < 0.05, ^**^P < 0.01, vs High group

To further investigate whether GPR34 is the key receptor for ZG-I to regulate ILC3s, we conducted experiments in vitro for verification. After successfully knocking down GPR34 in ILC3s, we co-cultured ILC3s with mouse fibroblasts (Fig. 7A–C). MTT assay showed that si-GPR34 significantly inhibited the viability of primary mouse skin fibroblasts (Fig. 7D). Detection of the proliferation and migration status of fibroblasts revealed that, compared to High group, both EDU⁺ cells counts and the number of migration cells/field were significantly reduced in si-GPR34 + High group. This indicates that GPR34 knockdown antagonized the promoting effects of ZG-I (Fig. 7E–H). The level of P-STAT3/STAT3 in si-GPR34 + High group was also inhibited in comparison with High group (Fig. 7I, J). Additionally, in Fig. 7K–N, the flow cytometry analysis demonstrated a decrease in the percentage of IL-22^+^ILC3s and P-STAT3^+^ILC3s in si-GPR34 + High group due to the GPR34 inhibition. The observed results confirmed that silencing GPR34 successfully counteracts the impact of ZG-I in vitro models.

### GPR34 is a therapeutic target for ZG-I

**Fig. 8:**
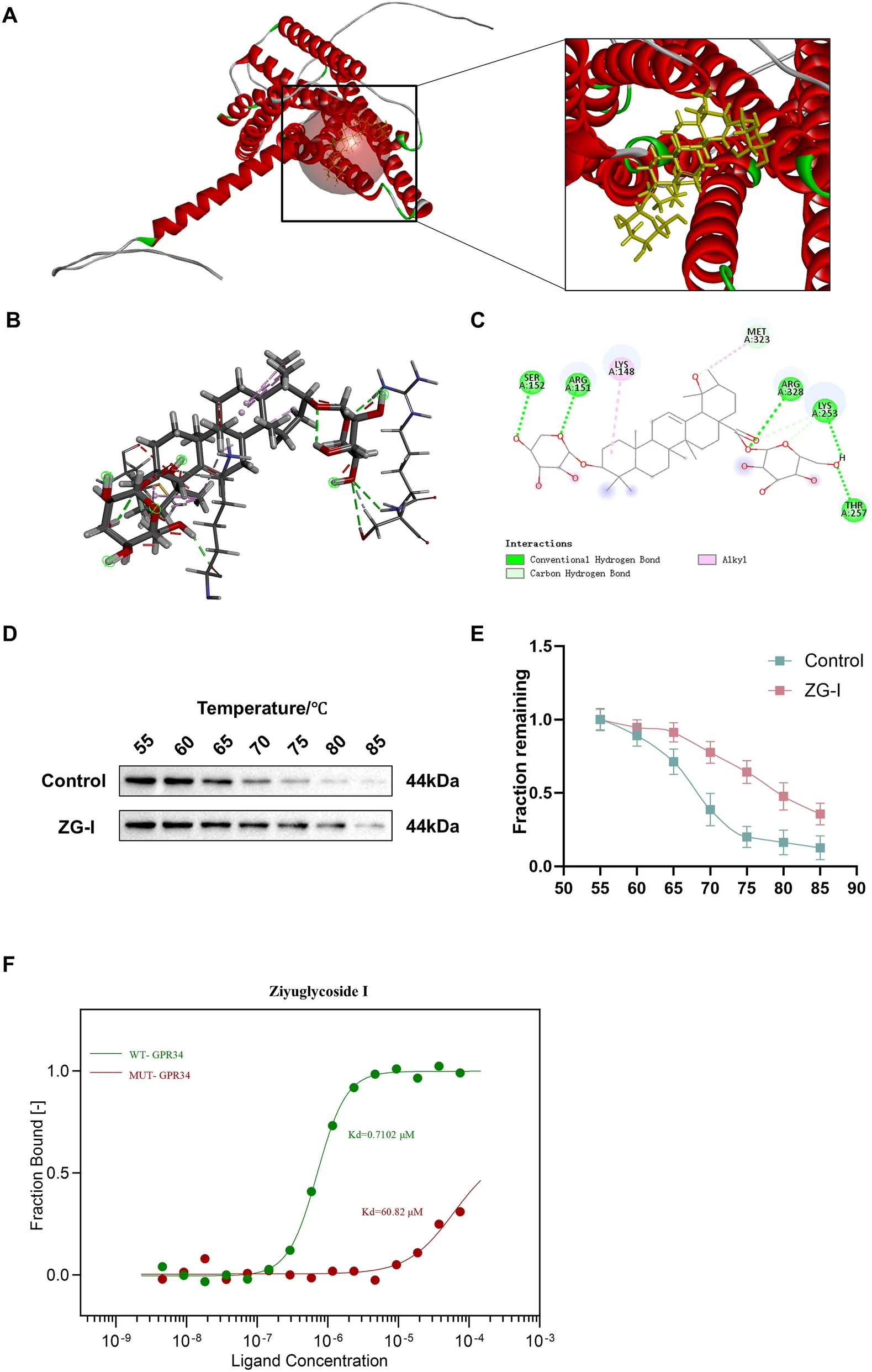
Verification of GPR34 as a ZG-I therapeutic target by molecular docking and CETSA experiment. **A** Schematic representation of the binding mode between ZG-1 and its target protein. The enlarged view on the right illustrates the detailed interactions at the active site. **B** Detailed interactions between ZG-I and key amino acid residues of the target protein, with dashed lines indicating hydrogen bonds and other non-covalent forces. **C** The interaction types and specific amino acid residues involved in the binding between ZG-I and the target protein, including conventional hydrogen bonds, carbon-hydrogen bonds, and alkyl interactions. **D** SDS-PAGE analysis of thermal stability. The gel image shows the changes in the 44 kDa protein band intensity for the Control and ZG-I-treated groups across different temperatures. **E** Quantitative analysis of thermal stability. The curve plots the remaining fraction of the target protein against temperature. **F** Binding affinity analysis of ZG-I to GPR34. The graph displays the fraction bound of ZG-I to WT-GPR34 (green) and MUT-GPR34 (red) as a function of ligand concentration. The Kd values are indicated: Kd = 0.7102 μM for WT-GPR34 and Kd = 60.82 μM for MUT-GPR34

The interaction between ZG-I (ligand) and mouse-derived GPR34 (receptor) was evaluated based on the LibDockScore, a key indicator of binding affinity in molecular docking. Predicted potential active pockets of mouse GPR34 include sites 187, 559, 647, 477, and 623. During the docking process, the small molecule may bind to multiple active pockets, generating multiple LibDockScores. A higher LibDockScore indicates a greater likelihood of ligand binding to the specific active pocket, stronger protein–ligand interaction, and higher biological activity. Therefore, the highest LibDockScore was selected as the primary metric to assess the docking performance. As shown in Table 2, the LibDockScore for active pocket 559 of mouse GPR34 with ZG-I was the highest (135.468), suggesting it as the most favorable binding site. The binding mode between mouse GPR34 and ZG-I is illustrated in Fig. 8A–C. Key molecular interactions, such as π-alkyl interactions and C-H bonds, contribute significantly to the strong binding affinity between ZG-I and mouse GPR34.

**Table 2:** Docking score of GPR34 and ZG-I

Pocket number	LibDockScore
187	102.731
559	135.468
647	103.953
477	103.296
623	122.463

The CETSA results demonstrated that ZG-I treatment significantly enhanced the thermal stability of GPR34 in a dose-dependent manner. This was observed as a rightward shift in the GPR34 melting curve (Fig. 8D, E), indicating that a higher temperature was required to denature GPR34 in the presence of ZG-I compared to the vehicle control. This ligand-induced thermal stabilization provides direct biophysical evidence that ZG-I physically engages with and binds to GPR34 within the cellular environment. These data substantially strengthen the conclusion that GPR34 is a direct therapeutic target of ZG-I.

To further quantify the direct binding affinity and validate the predicted binding site, MST assays were performed (Fig. 8F). The results revealed that ZG-I bound to WT-GPR34 with a high affinity, exhibiting a dissociation constant (Kd) of 0.71 µM. To confirm the specificity of the interaction for the predicted active pocket (site 559), a mutant GPR34 protein (MUT-GPR34) with key amino acid residues altered was generated. The binding affinity between ZG-I and MUT-GPR34 was significantly diminished, with the Kd value increasing to 60.82 µM. This substantial reduction in affinity provides compelling biophysical evidence that ZG-I directly and specifically binds to the predicted active pocket of GPR34. Collectively, the MST data, corroborating the molecular docking and CETSA findings, offer an evidence establishing GPR34 as a direct target of ZG-I.

## Discussion

Wound healing and tissue regeneration is an intricate biological process. It requires tight orchestration of cell migration, proliferation, matrix deposition and remodeling, alongside inflammation and angiogenesis [21]. Post-injury inflammation exhibits triphasic progression that orchestrates tissue repair. Initially, local chemotactic factors recruit key inflammatory cells during an early proinflammatory stage. Subsequently, these cells undergo phenotypic switching toward reparative functions, followed by their systematic elimination via apoptosis—particularly evident in neutrophils—to resolve the response and restore architectural integrity [22]. Recent study demonstrates that ILC3s contribute to orchestrating normal skin repair [9, 23, 24]. ILC3 facilitates epithelial cell proliferation and regeneration via IL-22 secretion [25–27]. Among them, GPR34 is critical for maintaining the therapeutic efficacy of ILC3s during physiological recovery from epithelial damage.

*Sanguisorba officinalis* L., a traditional Chinese medicinal herb historically employed for managing skin injuries and burns, has garnered significant attention in modern pharmacological research due to its multifaceted bioactive properties [14]. Experimental evidence supports their broader therapeutic potential across multiple disease models—such as anticancer activity against malignant cells, inhibitory effects on viral replication, bacterial growth suppression, neuroprotection in neural injury paradigms, and hepatoprotection through mitigation of liver damage induced by toxins or ischemia [28–32]. These findings collectively highlight the multifaceted medicinal value of the subject matter across diverse pathophysiological contexts. Concurrently, other taxa within the genus Sanguisorba are subjects of ongoing global research efforts aimed at elucidating their pharmacological profiles. These findings underscore the plant’s dual significance as a culturally rooted remedy and a promising candidate for developing novel therapeutic agents [15].

Recent studies demonstrate that neutrophils, upon apoptosis, release LysoPS, which acts as a ligand for GPR34, a receptor highly expressed on ILC3s. This interaction triggers a signaling cascade involving AKT and MAPK pathways, culminating in the robust production of IL-22, a cytokine pivotal for tissue repair and regeneration [10]. Blocking the downstream signaling cascades of GPR34 reduces both apoptotic neutrophil activity and LysoPS-mediated IL-22 synthesis [10]. Ziyuglycoside I is an important active component of *Sanguisorba officinalis* L., which has excellent pharmacological effects. Our vivo experimental studies demonstrated that ZG-I accelerated wound healing rates in mice and upregulated the expression of GPR34 and IL-22 in ILC3s from murine skin tissues. ZG-I does have the activity of promoting wound healing. Subsequently, we investigated the downstream signaling mechanisms of GPR34 associated with ILC3s activation. The transcription factor STAT3 serves as a central regulator of IL-22 production in ILC3s [33], a mechanism critical for maintaining epithelial integrity and modulating immune responses. While properly regulated STAT3 signaling supports infection defense and metabolic homeostasis, dysregulated STAT3 activity in ILC3s can exacerbate inflammation, highlighting its dual role in both physiological balance and inflammatory disease pathogenesis [34–36]. Our study demonstrates that GPR34 activates the AKT and MAPK signaling pathways in ILC3s, leading to the phosphorylation and activation of STAT3. This results in increased IL-22 production, as evidenced by elevated P-STAT3 levels and its co-localization with RORγt. These findings highlight the essential role of GPR34-mediated signaling pathways, specifically the AKT, MAPK, and STAT3 axes, in regulating ILC3 function and IL-22 expression. We also conducted in vitro experiments. After concentration screening, we co-cultured ILC3s treated with different concentrations of ZG-I along with mouse primary fibroblasts to further validate these results. Based on the given context, the function of ZG-I can be inferred as promoting the activation effect of neutrophils on ILC3 by enhancing the expression of GPR34 and the downstream cascade in ILC3 cells, thereby releasing IL-22, facilitating wound healing. To verify this mechanism, we knocked down GPR34 in the extracted murine ILC3s. This manipulation reversed the promoting effects of ZG-I on the proliferation and migration of fibroblasts, and also restored the expression levels of AKT and MAPK signaling cascades, as well as STAT3 and IL-22, to their baseline states observed in untreated conditions. Finally, our molecular docking analysis revealed that ZG-I directly binds to GPR34, with the strongest interaction occurring at the receptor’s active pocket 559, primarily mediated by π-alkyl interactions and C-H bonds. This interaction was further validated using the CETSA and MST. These findings collectively establish GPR34 as a direct target of ZG-I, supporting its potential for further therapeutic development.

Several limitations of this study warrant careful consideration. Firstly, while our in vitro experiments provide strong evidence that ZG-1 exerts its regulatory effects on ILC3 function by activating multiple independent signaling branches downstream of GPR34, specifically the AKT, MAPK, and STAT3 axes, the in vivo physiological significance of these signaling events has not yet been comprehensively established. For instance, the use of ILC3-specific or systemic GPR34 knockout mouse models would be essential to confirm whether ZG-I's wound-healing effects are entirely dependent on GPR34 in vivo, as the complex tissue microenvironment may harbor alternative or compensatory signaling pathways. Secondly, our current investigation into ILC3 crosstalk has been confined to interactions with neutrophils and fibroblasts, leaving its potential regulatory effects on other critical immune players—such as macrophage polarization or T lymphocyte function—largely unexplored. A more comprehensive understanding of how ZG-I influences the broader immune network would significantly enhance our mechanistic insight. Thirdly, while GPR34 has been identified as a target, the possibility of ZG-I acting on additional molecular targets cannot be excluded, which may contribute to its overall therapeutic effect. Moreover, the clinical translatability of our findings is limited by the use of a standardized acute full-thickness wound model in healthy mice, which may not fully recapitulate the pathological microenvironment of chronic, non-healing wounds (e.g., diabetic ulcers) characterized by persistent inflammation, ischemia, and infection. Finally, our study lacks long-term safety and efficacy data, including pharmacokinetic profiling, tissue functional recovery assessment, and potential adverse reactions, which are critical for evaluating the translational potential of ZG-I as a therapeutic agent.

## Conclusion

Collectively, our results indicate that ZG-I has the activity of promoting wound healing, and GPR34 can serve as a therapeutic target for ZG-I in the treatment of skin injury-related diseases.

## Data Availability

No datasets were generated or analysed during the current study.

## Abbreviations

Akt: Protein kinase B
ECM: Extracellular matrix
ERK: Extracellular regulated protein kinases
GPR34: G protein-coupled receptor 34
IL-22: Interleukin-22
ILCs: Innate lymphoid cells
ILC3s: Group 3 innate lymphoid cells
LysoPS: Lysophosphatidylserine
MAPK: Mitogen-activated protein kinase
RORγ: Retinoic acid receptor-related orphan receptor γ
STAT3: Signal transducer and activator of transcription 3
ZG-I: Ziyuglycoside I

## References

[1] Plikus MV, Gay DL, Treffeisen E, Wang A, Supapannachart RJ, Cotsarelis G. Epithelial stem cells and implications for wound repair. Semin Cell Dev Biol. 2012;23:946–53. 10.1016/j.semcdb.2012.10.001. 23085626 10.1016/j.semcdb.2012.10.001 PMC3518754

[2] Fitridge R, Thompson M. Mechanisms of vascular disease: a reference book for vascular specialists. 1st ed. Adelaide: University of Adelaide Press; 2011. 10.1017/UPO9781922064004. 30484990

[3] Lothstein KE, Chen F, Mishra P, Smyth DJ, Wu W, Lemenze A, et al. Helminth protein enhances wound healing by inhibiting fibrosis and promoting tissue regeneration. Life Sci Alliance. 2024;7:e202302249. 10.26508/lsa.202302249. 39179288 10.26508/lsa.202302249 PMC11342954

[4] Sarate RM, Hochstetter J, Valet M, Hallou A, Song Y, Bansaccal N, et al. Dynamic regulation of tissue fluidity controls skin repair during wound healing. Cell. 2024;187:5298-5315.e19. 10.1016/j.cell.2024.07.031. 39168124 10.1016/j.cell.2024.07.031

[5] Falanga V. Wound healing and its impairment in the diabetic foot. Lancet. 2005;366:1736–43. 10.1016/S0140-6736(05)67700-8. 16291068 10.1016/S0140-6736(05)67700-8

[6] Artis D, Spits H. The biology of innate lymphoid cells. Nature. 2015;517:293–301. 10.1038/nature14189. 25592534 10.1038/nature14189

[7] Serafini N, Vosshenrich CAJ, Di Santo JP. Transcriptional regulation of innate lymphoid cell fate. Nat Rev Immunol. 2015;15:415–28. 10.1038/nri3855. 26065585 10.1038/nri3855

[8] Eberl G, Colonna M, Di Santo JP, McKenzie ANJ. Innate lymphoid cells: a new paradigm in immunology. Science. 2015;348:aaa6566. 10.1126/science.aaa6566. 25999512 10.1126/science.aaa6566 PMC5658207

[9] Li Z, Hodgkinson T, Gothard EJ, Boroumand S, Lamb R, Cummins I, et al. Epidermal Notch1 recruits RORγ+ group 3 innate lymphoid cells to orchestrate normal skin repair. Nat Commun. 2016;7:11394. 10.1038/ncomms11394. 27099134 10.1038/ncomms11394 PMC4844683

[10] Wang X, Cai J, Lin B, Ma M, Tao Y, Zhou Y, et al. GPR34-mediated sensing of lysophosphatidylserine released by apoptotic neutrophils activates type 3 innate lymphoid cells to mediate tissue repair. Immunity. 2021;54:1123-1136.e8. 10.1016/j.immuni.2021.05.007. 34107271 10.1016/j.immuni.2021.05.007

[11] Arshad T, Mansur F, Palek R, Manzoor S, Liska V. A double edged sword role of interleukin-22 in wound healing and tissue regeneration. Front Immunol. 2020;11:2148. 10.3389/fimmu.2020.02148. 33042126 10.3389/fimmu.2020.02148 PMC7527413

[12] Sabat R, Ouyang W, Wolk K. Therapeutic opportunities of the IL-22–IL-22R1 system. Nat Rev Drug Discov. 2014;13:21–38. 10.1038/nrd4176. 24378801 10.1038/nrd4176

[13] Zaharie RD, Popa C, Schlanger D, Vălean D, Zaharie F. The role of IL-22 in wound healing. Potential implications in clinical practice. Int J Mol Sci. 2022;23:3693. 10.3390/ijms23073693. 35409053 10.3390/ijms23073693 PMC8998254

[14] Song J, Zeng J, Zheng S, Jiang N, Wu A, Guo S, et al. *Sanguisorba officinalis* L. promotes diabetic wound healing in rats through inflammation response mediated by macrophage. Phytother Res. 2023;37:4265–81. 10.1002/ptr.7906. 37260161 10.1002/ptr.7906

[15] Zhou P, Li J, Chen Q, Wang L, Yang J, Wu A, et al. A comprehensive review of genus *Sanguisorba*: traditional uses, chemical constituents and medical applications. Front Pharmacol. 2021;12:750165. 10.3389/fphar.2021.750165. 34616302 10.3389/fphar.2021.750165 PMC8488092

[16] Melzig MF. Der Große Wiesenknopf Sanguisorba officinalis L. Z Phytother. 2024;45:41–7. 10.1055/a-2171-9505.

[17] Zhong Y, Tian X, Jiang X, Dang W, Cheng M, Li N, et al. Novel Ziyuglycoside II derivatives inhibit MCF-7 cell proliferation via inducing apoptosis and autophagy. Bioorg Chem. 2023;139:106752. 10.1016/j.bioorg.2023.106752. 37499529 10.1016/j.bioorg.2023.106752

[18] Kim YH, Chung CB, Kim JG, Ko KI, Park SH, Kim J-H, et al. Anti-wrinkle activity of Ziyuglycoside I isolated from a *Sanguisorba officinalis* root extract and its application as a cosmeceutical ingredient. Biosci Biotechnol Biochem. 2008;72:303–11. 10.1271/bbb.70268. 18256460 10.1271/bbb.70268

[19] Lee H-S, Kim B-K, Lee S-Y, Kwon H, Park H-W. Essential role of Card11 in airway hyperresponsiveness in high-fat diet-induced obese mice. Exp Mol Med. 2024;56:2747–54. 10.1038/s12276-024-01367-z. 39672814 10.1038/s12276-024-01367-z PMC11671588

[20] Chun E, Lavoie S, Fonseca-Pereira D, Bae S, Michaud M, Hoveyda HR, et al. Metabolite-sensing receptor Ffar2 regulates colonic group 3 innate lymphoid cells and gut immunity. Immunity. 2019;51:871-884.e6. 10.1016/j.immuni.2019.09.014. 31628054 10.1016/j.immuni.2019.09.014 PMC6901086

[21] Peña OA, Martin P. Cellular and molecular mechanisms of skin wound healing. Nat Rev Mol Cell Biol. 2024;25:599–616. 10.1038/s41580-024-00715-1. 38528155 10.1038/s41580-024-00715-1

[22] Eming SA, Wynn TA, Martin P. Inflammation and metabolism in tissue repair and regeneration. Science. 2017;356:1026–30. 10.1126/science.aam7928. 28596335 10.1126/science.aam7928

[23] Aparicio-Domingo P, Romera-Hernandez M, Karrich JJ, Cornelissen F, Papazian N, Lindenbergh-Kortleve DJ, et al. Type 3 innate lymphoid cells maintain intestinal epithelial stem cells after tissue damage. J Exp Med. 2015;212:1783–91. 10.1084/jem.20150318. 26392223 10.1084/jem.20150318 PMC4612094

[24] Zhang W, Mu X, Hang Q, Huang Y, Ding Y, Xu T, et al. Role of group 3 innate lymphoid cells in skin wound healing and underlying mechanisms. Chin J Dermatol. 2024;57:516–23. 10.35541/cjd.20230676.

[25] Saez De Guinoa J, Jimeno R, Farhadi N, Jervis PJ, Cox LR, Besra GS, et al. CD 1d‐mediated activation of group 3 innate lymphoid cells drives IL‐22 production. EMBO Rep. 2017;18:39–47. 10.15252/embr.201642412. 27799287 10.15252/embr.201642412 PMC5210076

[26] Pandey A, Bansal A, DeJesus E, Kisiel G, Clark WA, Skawinski M, et al. 429 differentiation of IL-22 levels in inflammatory diseases using a high sensitivity ELISA. Br J Dermatol. 2023;188:ljad162.049. 10.1093/bjd/ljad162.049.

[27] Romera-Hernández M, Aparicio-Domingo P, Papazian N, Karrich JJ, Cornelissen F, Hoogenboezem RM, et al. Yap1-driven intestinal repair is controlled by group 3 innate lymphoid cells. Cell Rep. 2020;30:37-45.e3. 10.1016/j.celrep.2019.11.115. 31914395 10.1016/j.celrep.2019.11.115

[28] Yasueda A, Kayama H, Murohashi M, Nishimura J, Wakame K, Komatsu K, et al. *Sanguisorba officinalis* L. derived from herbal medicine prevents intestinal inflammation by inducing autophagy in macrophages. Sci Rep. 2020;10:9972. 10.1038/s41598-020-65306-4. 32561763 10.1038/s41598-020-65306-4 PMC7305163

[29] Wang T, Tian X-L, Xu X-B, Li H, Tian Y, Ma Y-H, et al. Dietary supplementation of probiotics fermented Chinese herbal medicine *Sanguisorba officinalis* cultures enhanced immune response and disease resistance of crucian carp (*Carassius auratus*) against *Aeromonas hydrophila*. Fish Shellfish Immunol. 2022;131:682–96. 10.1016/j.fsi.2022.10.046. 36341871 10.1016/j.fsi.2022.10.046

[30] Jiang N, Li H, Sun Y, Zeng J, Yang F, Kantawong F, et al. Network pharmacology and pharmacological evaluation reveals the mechanism of the *Sanguisorba officinalis* in suppressing hepatocellular carcinoma. Front Pharmacol. 2021;12:618522. 10.3389/fphar.2021.618522. 33746755 10.3389/fphar.2021.618522 PMC7969657

[31] Long W, Liu S, Li X-X, Shen X, Zeng J, Luo J-S, et al. Whole transcriptome sequencing and integrated network analysis elucidates the effects of 3,8-Di-O-methylellagic acid 2-O-glucoside derived from *Sanguisorba offcinalis* L., a novel differentiation inducer on erythroleukemia cells. Pharmacol Res. 2021;166:105491. 10.1016/j.phrs.2021.105491. 33582247 10.1016/j.phrs.2021.105491

[32] Chen P, Chen M, Peng C, Yan J, Shen X, Zhang W, et al. In vitro anti-bactrical activity and its preliminary mechanism of action of the non-medicinal parts of *Sanguisorba officinalis* L. against *Helicobacter pylori* infection. J Ethnopharmacol. 2024;318:116981. 10.1016/j.jep.2023.116981. 37574016 10.1016/j.jep.2023.116981

[33] Rutz S, Wang X, Ouyang W. The IL-20 subfamily of cytokines — from host defence to tissue homeostasis. Nat Rev Immunol. 2014;14:783–95. 10.1038/nri3766. 25421700 10.1038/nri3766

[34] Xi X, Hu R, Wang Q, Xu K, Yang H, Cui Z, et al. Interleukin‑22 promotes PD‑L1 expression via STAT3 in colon cancer cells. Oncol Lett. 2021;22:716. 10.3892/ol.2021.12977. 34429756 10.3892/ol.2021.12977 PMC8371963

[35] Zhou J, Yue J, Yao Y, Hou P, Zhang T, Zhang Q, et al. Dihydromyricetin protects intestinal barrier integrity by promoting IL-22 expression in ILC3s through the AMPK/SIRT3/STAT3 signaling pathway. Nutrients. 2023;15:355. 10.3390/nu15020355. 36678226 10.3390/nu15020355 PMC9861697

[36] Bao B, Wang Y, Boudreau P, Song X, Wu M, Chen X, et al. Bacterial sphingolipids exacerbate colitis by inhibiting ILC3-derived IL-22 production. Cell Mol Gastroenterol Hepatol. 2024;18:101350. 10.1016/j.jcmgh.2024.04.007. 38704148 10.1016/j.jcmgh.2024.04.007 PMC11222953

